# Physical mechanisms of oceanic mantle earthquakes: Comparison of natural and experimental events

**DOI:** 10.1038/s41598-018-35290-x

**Published:** 2018-11-19

**Authors:** Saeko Kita, Thomas P. Ferrand

**Affiliations:** 1 Graduate School of Science, Hiroshima University, Kagamiyama1-3-1, Higashi, Hiroshima, 739-8526 Japan; 2 Present Address: Building Research Institute, Tatehara 1, Tsukuba, 305-0802 Japan; 3 Laboratoire de Géologie – UMR 8538, CNRS, Ecole Normale Supérieure, PSL University, 75005 Paris, France; 4 Earthquake Research Institute, University of Tokyo, 1-1-1 Yayoi, Bunkyo-ku, Tokyo, 113-0032 Japan

## Abstract

Because they provide information about the spatial distribution of brittle deformation, both seismologists and experimentalists use b-values to study earthquake populations. Here, we present the b-values for intermediate-depth intraslab earthquakes in the Pacific slab beneath the Tohoku and Hokkaido regions, northeastern Japan and find a difference in the lower-plane event b-values in the double seismic zone. Lower-plane events reveal significantly larger b-values beneath Tohoku (0.96) than Hokkaido (0.86), implying that the brittle deformation beneath Hokkaido is more localized and leads to higher ratio of relatively large lower-plane events than occur beneath Tohoku. We also estimated the b-values for experimental earthquakes, and found they increase with increasing antigorite content in serpentinized peridotite. These experimental earthquakes already led to the “dehydration driven stress transfer” (DDST) model, which suggests that a highly hydrated peridotite is not required when oceanic mantle events occur. A comparison of experimental and natural earthquake b-values implies that lower-plane peridotite is more hydrated beneath the Tohoku region, which could also explain the difference in oceanic-plate velocity structures near the trench identified in Ocean Bottom Seismometer studies off Tohoku and Hokkaido. These results suggest that lower-plane events occur in fresh peridotite near serpentinized faults.

## Introduction

In seismology, b-value analyses provide an important tool for understanding the nature of earthquakes. The b-value is the slope of the Gutenberg-Richter frequency-magnitude distribution. Specifically, the b-value is defined by log_10_*N* = *a* − *b* *M*^min^, where *N* is the cumulative number of earthquakes with a magnitude *M* higher than *M*^min^, and a is the number of events with *M* ≥ 0. Results of laboratory earthquake studies^1^ and observational studies^2^ have shown that more heterogeneous materials lead to higher b-values. Beneath northeastern Japan, b-values have been extensively studied^3–7^.

**Figure 1:**
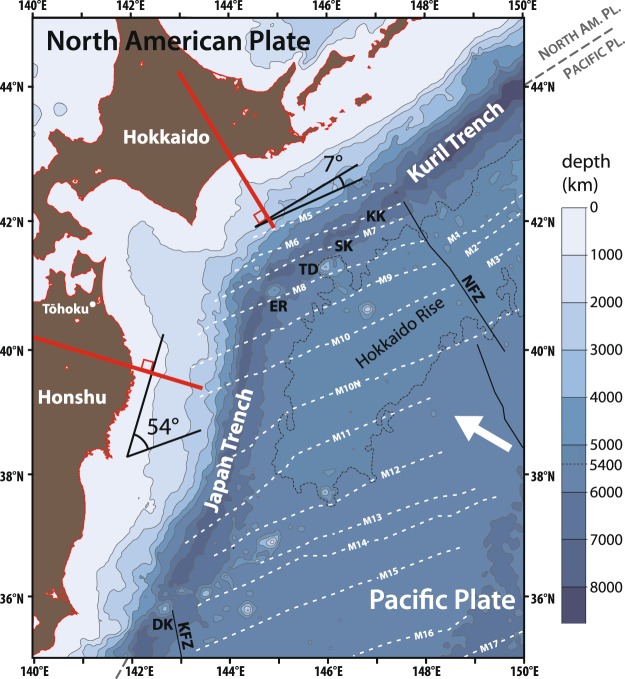
Bathymetric map (modified^7^) showing locations of the trench axis (>7000 m) and outer swell (Hokkaido Rise <5400 m) together with magnetic isochrones (white dashed lines) and fracture zones in the northwestern Pacific margin. The direction of plate convergence is denoted by a thick arrow. Plate motion is recalculated using the model NUVEL-1 (relative motion, Pacific plate fixed)^8^: 86.42 mm year^−1^ with azimuth N299.6E at 38.4 N, 149.75E (*V*_lat_ = 42.63 mm year^−1^, *V*_lon_ = −75.18 mm year^−1^). KFZ: Kashima Fault Zone, NFZ: Nosappu Fault Zone, TD: Takuyo-Daiichi Seamount, ER: Erimo Seamount, DK: Daiichi-Kashima Seamount, SK: Mashu Knoll, KK: Kamuishu Knoll.

In northeastern Japan, the Pacific plate subduct beneath the North American Plate at the Kuril Trench in the Hokkaido region and at the Japan Trench in the Tohoku region. Although the age of the Pacific is almost the same in both regions, the subduction is oblique in eastern Hokkaido due to a bend in the boundary of the Japan and Kuril trenches axes (Fig. 1). The magnetic isochrones offshore of northeastern Japan are subparallel to the Kuril Trench where it makes a high angle with the Japan Trench (Fig. 1)^8,9^. The preexisting spreading fabric drive the final hydration level^10^ and may control the earthquake distribution for the entire subducting slab. This difference in trench orientation between the Hokkaido and Tohoku regions makes northeastern Japan a very interesting region for studies to understand the influence of the spreading faults, which could be a key to unraveling earthquake nucleation processes.

Beneath northeastern Japan, intermediate-depth seismicity in the subducting oceanic plate (Pacific Plate) is very active, and many studies have revealed characteristics of the regional seismicity. The spatial distribution of the intraslab earthquakes beneath the Tohoku^11^ and Hokkaido^12^ regions was examined, revealing that the Wadati-Benioff zone actually consists of a double seismic structure for all subduction zones^13^. The focal mechanisms in such double seismic zones^11,12,14^ indicate that the upper plane yields in the down-dip compression stress regime while the lower plane undergoes down-dip extension.

**Figure 2:**
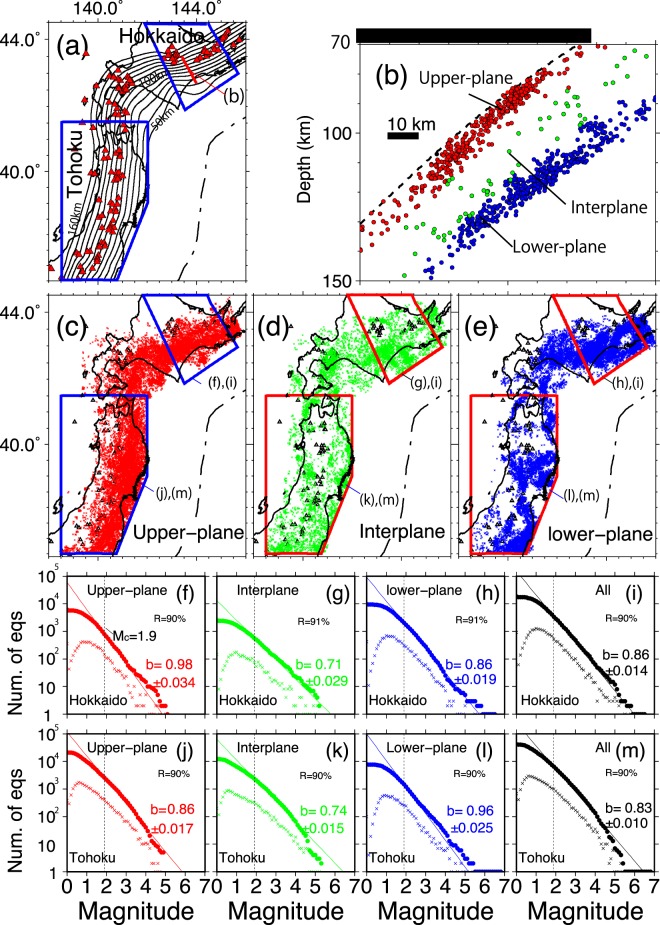
(**a**) Spatial distribution of the depth contours of the upper plate interface of the subducting Pacific Plate^15^. Sub-areas used in this study are shown as blue polygons. (**b**) Vertical cross-section showing the relocated hypocenters beneath Hokkaido along the red profile shown in Fig. 2a. (**c**–**e**) Spatial distributions of the three groups of relocated intraslab earthquakes, as explained in text. In (**a)** to (**e**) red, green and blue dots refer to upper-plane, interplane and lower-plane earthquakes, respectively. (**f**–**j**) Gutenberg-Richter distributions for upper-plane, interplane, lower-plane and entire intraslab events beneath Hokkaido. (**j**–**m**) Gutenberg-Richter maps for upper-plane, interplane, lower-plane, and entire intraslab events beneath the Tohoku region. In **(f) to (m)**, dots and crosses show the cumulative number of events and the number of events, respectively. The thin straight lines in **(f)** to **(m)** show the calculated cumulative number of events.

For decades, the physical mechanisms of intermediate-depth earthquakes have been a matter of debate because, when compared to thrust and inland type earthquakes, their triggering mechanisms are puzzling. Lately, the understanding of the double seismic structure has progressed based on examinations of precisely relocated intraslab events using the Japanese nationwide dense seismic network data. Oceanic crust events were relocated beneath northeastern Japan and their spatial distribution was examined^15^. Seismicity peaks located about 80 km deep were inferred to be correlated with metamorphic reactions due to MORB dehydration, which implies that dehydration embrittlement is a possible triggering mechanism for intermediate-depth oceanic crust events. Steady seismicity, i.e. excluding aftershocks of large events, was also revealed between the two planes of the double seismic zone (Figs. 2a, 2b and 2d).

Within a subducting slab, the physical mechanisms of oceanic mantle events, i.e. lower-plane and interplane events, are not as easy to imagine as those for events occurring near the subduction interface, i.e. upper-plane events. Several hypotheses for the triggering process for oceanic mantle events at intermediate depth have been proposed^16–18^. Recently, synthetic serpentinized peridotites, i.e. olivine-antigorite aggregates, were deformed at high pressure using D-DIA^19^, and examined by combining synchrotron and acoustic-emission technologies in order to gain a better understanding of the intermediate-depth earthquake generation process in the mantle^18^. In such synthetic serpentinized peridotite, a dehydration-driven stress transfer (DDST)^18^ has been proposed as a possible mechanism for triggering intermediate-depth seismicity. The DDST model has recently been supported by field observations in peridotites^20–22^, whereas the shear of dehydrating antigorite is aseismic under the same conditions^23–26^. In the DDST hypothesis, the generation mechanism for intermediate-depth oceanic mantle earthquakes has a strain localization triggering stage, followed by a strain localization acceleration stage, and then the earthquake occurrence stage. In contrast, the acceleration stage of strain localization is not included in the “dehydration-embrittlement” hypothesis^27^.

Although several authors have studied b-values for intraslab earthquakes beneath northeastern Japan^3,4,7,28,29^, they did not examine the difference in b-values for the intermediate-depth events between the Tohoku and Hokkaido regions. Accordingly, in this study, we examined the b-values for the intraslab earthquakes beneath Tohoku and Hokkaido using precisely relocated hypocenters and the detailed geometry of the upper interface of the Pacific Plate. We also calculated the b-values for experimental earthquakes and compared them with those for natural earthquakes in the oceanic mantle (mainly lower-plane events) in order to better constrain the nature of lower-plane earthquakes, and to more fully understand the physical mechanisms for subducting oceanic mantle earthquakes.

## Results

### A precise relocation in three groups

We relocated 86,655 hypocenters at depths of 60 to 200 km from June 2002 to September 2016 beneath the Tohoku and eastern Hokkaido regions using double difference relocation^30^. The upper limit of the depth interval is fixed at 60 km because the lower limit of low angle thrust type events is ≈55 km beneath the Tohoku region and ≈60 km beneath the eastern Hokkaido region^31–33^. The three-dimensional (3D) spatial distributions (geographical position and depth) of the relocated hypocenters that were used in our examinations are shown in Fig. S1.

Next, we calculated b-values beneath the Tohoku and eastern Hokkaido regions, the areas of which are shown in Fig. 2. The western edge of the eastern Hokkaido region corresponds to the sharp corner in the depth contours of the geometry of the Pacific Plate upper interface beneath the Hidaka collision zone^34^ (Figs. 2a, 2c and 2e). For our examinations, intraslab earthquakes were also divided them into three types (Fig. 2b) using their normal distance from the subduction interface^34^ (Fig. 2a): upper-plane events (0–10 km from the subduction interface; Fig. 2c), interplane events (in between the upper and lower seismic planes, 10–23 km; Fig. 2d) and the lower plane events (more than 23 km from the subduction interface; Fig. 2e). The position of the subduction interface was precisely estimated^33^ using the geometry deduced from converted waves^35^, hypocenter locations, repeating earthquakes, and low-angle thrust earthquakes.

### Gutenberg-Richter distributions show significant differences in b-values

Magnitude frequency distributions of events beneath all of northeastern Japan, the Tohoku region and the Hokkaido region are respectively shown in Figs. 2f to 2m, along with their associated calculated b-values and errors. Events beneath the Tohoku region before and after the *M*9 event are calculated separately. The b-value for the entire slab beneath the Tohoku region (0.83 ± 0.010) is almost the same as that beneath the Hokkaido region (0.86 ± 0.015). Figure 2 and Table 1 show b-values for three types of intraslab events (upper-plane events, interplane events, and lower-plane events) beneath the eastern Hokkaido and Tohoku regions.

**Table 1:** Calculated b-values and associated errors beneath the Tohoku and eastern Hokkaido regions using *M*_c_ = 1.9.

Location	TOHOKU				HOKKAIDO	
	before M9		>6 months after M9			
	b-value	nb EQs	b-value	nb EQs	b-value	nb EQs
entire slab	0.83 ± 0.010	6443	0.82 ± 0.017	2357	0.86 ± 0.015	3430
upper plane	0.86 ± 0.017	2608	0.80 ± 0.026	958	0.98 ± 0.035	798
interplane	0.74 ± 0.015	2379	0.71 ± 0.029	616	0.71 ± 0.030	580
lower plane	0.96 ± 0.025	1456	0.92 ± 0.033	783	0.86 ± 0.019	2052

The results are given for the entire slab between 60 and 200 km depth, as well as for each of the three earthquakes populations at those depths, consistently with Fig. 2. For the Tohoku region, the events before and after the *M*9 event are processed separately in order to examine their impact on intermediate-depth seismicity.

Beneath the Tohoku region, the b-values for the upper-plane events, interplane events, and lower-plane events are respectively 0.86 ± 0.017, 0.74 ± 0.015, and 0.96 ± 0.025 before the *M*9 event, and 0.80 ± 0.026, 0.71 ± 0.029, and 0.92 ± 0.033 six months after the *M*9 event. Beneath the Hokkaido region, the b-values for the upper-plane events, interplane events and lower-plane events are respectively 0.98 ± 0.035, 0.71 ± 0.030, and 0.86 ± 0.019. The b-value for the interplane events beneath the Tohoku region is low and almost the same as that beneath the eastern Hokkaido region, which indicates that seismic events are much more localized. In contrast, b-values beneath the Tohoku region are significantly higher in the lower plane and significantly smaller in the upper plane compared to the Hokkaido region. However, even taking into account of estimation errors, it is clear that these differences exist.

We also estimated b-values for smaller areas by further dividing the Tohoku and eastern Hokkaido (E-Hokkaido) regions into subareas in order to see whether or not a remarkable along-arc variation exists. Specifically, we split the Tohoku region into two or three smaller subareas and then conducted estimations, the results of which are shown in Tables S2a to S2d. In the Hokkaido region, the b-values for the upper-plane, lower-plane, and interplane events beneath the small subareas were almost the same as those beneath the entire Hokkaido region. In the Tohoku region, the b-value for the lower-plane and interplane events for all of the small subareas are stable, but the upper-plane b-values are unstable (0.78 to 1.03).

### Experimental b-values

**Figure 3:**
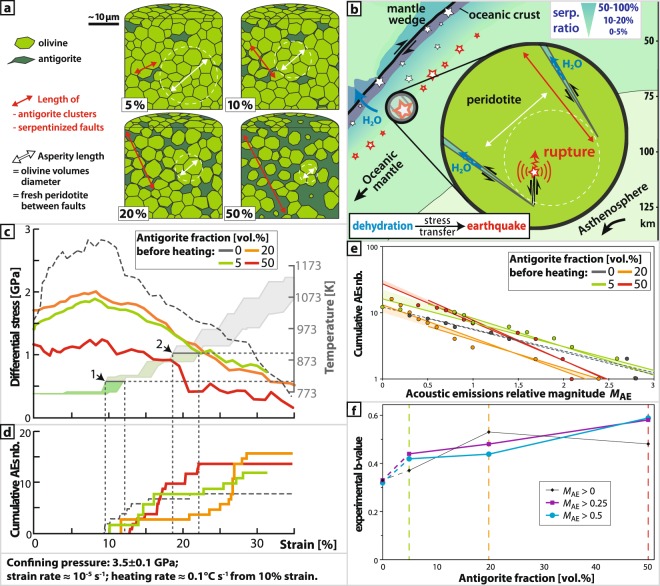
Comparison of the experiments at 3.5 GPa (≈115 km). (**a**) Elementary representative volume of olivine-antigorite samples used as a starting material for deformation experiments^16^ with 0, 5, 20, and 50% of antigorite (dark green); white arrows are mean diameter of stressed olivine volumes (light green), decreasing with increasing antigorite fraction; (**b**) sketch of two serpentinized fault tips in a subducting plate, representing upper-plane and interplane events (white stars) and focusing on lower-plane events (red stars); (**c**) stress and imposed temperature evolution as a function of strain^16^; (**d**) acoustic emissions cumulative number as a function of strain^16^; (**e**) Gutenberg-Richter distributions of acoustic emissions, with two calculations for two magnitudes of completeness, i.e. M_AE_ > 0 and M_AE_ > 0.5.; (**f**) experimental b-value as a function of initial antigorite volume fraction considering various magnitudes of completeness.

We estimated b-values for 79 acoustic emission (AE) events from four antigorite-olivine samples using the dataset of the DDST study^18^. The estimation method for b-values for experimental AE events is given in Table S2 and described in the Methods Section. These experimental b-values show an increase with increasing antigorite fraction (Figs. 3d and 3e; Table S2). The magnitude of these AE events decreases with increasing initial antigorite fraction, i.e. the hydration level (Fig. 3e). Though the fact that the number of AEs events is not so large could not be ignored, we consider that experimental b-values can be used to interpret b-values for natural earthquakes.

## Discussion

### The b-value for lower-plane events and its relationship with the hydration degree in the oceanic mantle

Lower-plane b-values are relatively stable beneath the entire Tohoku and entire eastern Hokkaido regions, although they are clearly larger beneath the Tohoku region (0.96) than beneath the eastern Hokkaido region (0.86). This discussion will focus primarily on discerning the cause of b-value differences for the lower-plane events of the Tohoku and Hokkaido regions by comparing the natural observational facts and knowledge gleaned from other studies and experimental results.

The difference in lower-plane b-value beneath Tohoku and Hokkaido is 0.1. On the other hand, the b-value errors of Tohoku and Hokkaido are small (±0.025 for Tohoku and ±0.019 for Hokkaido) because the number of events is large (1456 events for Tohoku and 2052 events for Hokkaido) and b-value error decreases with increasing number of events. Thus, the observed difference is not large, but exist.

The results of deformation experiments outlined in the DDST paper on slightly serpentinized artificial peridotites, i.e. antigorite-olivine aggregates, imply that a stress transfer from antigorite portions to olivine portions is the triggering mechanism for intermediate-depth oceanic mantle events^18^ (Figs. 3a to 3d). We estimated b-values for AE events in the samples of the DDST model, finding that these experimental b-values increase with increasing antigorite fractions (Figs. 3d and 3e; Table S2).

The DDST model does not require the oceanic mantle to be highly hydrated, but partly hydrated peridotite, most probably localized serpentinization, is needed at deep faults. The seismic tomography beneath the Hokkaido region^36^ showed a low P-wave velocity anomaly in the lower plane of the double seismic zone. Beneath the Tohoku-Oki regions, other seismic observations indicated the occurrence of intraplate earthquakes under an extensional stress field at depths of 0 to 40 km after the M9.0 event^37^, which possibly means that the subducting oceanic mantle at the depths of the lower-plane portion can be hydrated because the peak of lower plane events at intermediate-depth was located ~30 km from the upper plate interface^34^. In the DDST model, the oceanic mantle events do not necessarily occur on pre-existing structures, but instead take place in fresh peridotite near the serpentinized zones. We think that pre-existing structures that are hydrated in near-trench regions can be a source of fluid in the oceanic mantle at intermediate depths.

Observations in other subduction zones also show evidence of deep faults at subduction trenches^38–42^. Despite the absence of substantially reduced seismic velocities at 10 km and deeper^43^, recent seismic reflection surveys have revealed deep reflections that can be interpreted as bending-related faulting and mantle serpentinization at the Middle America Trench^38,39^ and offshore Alaska^40^. These observations indicate that the lithospheric mantle was partially hydrated 8 km and 15 km below the Moho, respectively, through serpentinization of deep faults, which may extend to 35 km depth based on an estimate of the brittle-ductile transition of the lithosphere^38^. Evidence of the direct link between mantle hydration and the generation of dehydration-induced intermediate-depth seismicity is also demonstrated offshore Alaska^40^.

Taking into consideration the experimental results and supporting evidence for a hydrated oceanic mantle, the difference of b-values in the lower-plane between the two regions could be related to the difference in the degree of hydration in the oceanic mantle. Oceanic mantle rocks where lower-plane events occur beneath the Tohoku region should be more hydrated than the oceanic mantle rocks of lower-plane events beneath the Hokkaido region. The results of our seismicity analysis imply that the subducting oceanic crust is more hydrated beneath the Tohoku region than beneath the Hokkaido region because the event numbers in the upper-plane seismic belt, i.e. the seismicity peak in oceanic crust at intermediate depths caused by MORB dehydration, is much higher beneath the Tohoku region^9,15,44^. The seismic velocity image beneath the offshore region using the Ocean Bottom Seismometers (OBS) also indicated that the reduction of the P-wave velocity in oceanic mantle beneath the Tohoku-Oki region is 5–10%, although it is only a few percent beneath the Hokkaido-Oki region. Moreover, the area showing the velocity reduction is much larger beneath the Tohoku-Oki region than beneath the Hokkaido-Oki region. As events in more heterogeneous material have a higher b-value^1,2^, our results for the lower plane suggest that the oceanic mantle is more heterogeneous beneath the Tohoku region than beneath the Hokkaido region, which is consistent with a higher degree of hydration at intermediate depths beneath the Tohoku region.

We think that the difference in the seismic velocity structures of the oceanic mantle beneath the Tohoku-Oki and Hokkaido-Oki regions that are indicated by the OBS campaigns^44^ may originate from the difference of hydration degree in the oceanic mantle. In this interpretation, we distinguish between two types of normal faults in the oceanic slab – bending faults that accommodate bending at the outer rise, and ‘spreading faults’ formed to accommodate extensional deformation at the spreading ridge. The geometric relationship between these two types of faults may be related to the degree of hydration of the subducting slab. The slab beneath the Tohoku-Oki region may be more hydrated than beneath the eastern Hokkaido region because of the strike differences between the spreading faults, i.e. the direction of magnetic anomalies, and the bending faults, i.e. the strike of the Japan and Kuril Trenches^8^ (Fig. 1).

**Figure 4:**
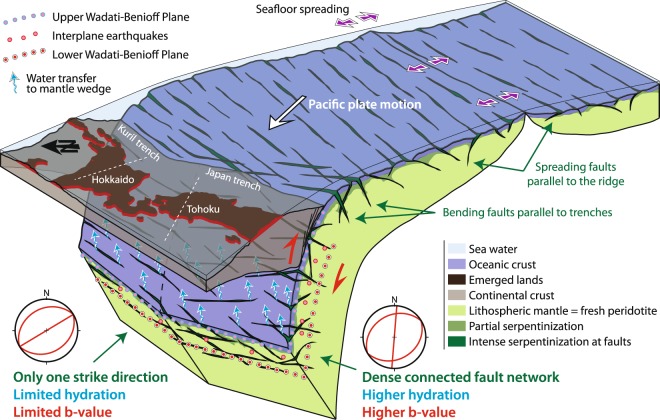
Sketch illustrating the link between spreading faults, fault networks connectivity, and seismicity in the lower plane of the double seismic zone. Upper stereographic projections show a clear distinction in the spatial distribution of faults beneath Hokkaido and Tohoku, with only one or two strike directions, respectively. Here, we represent deep serpentinized faults crossing the entire double-seismic zone. However, as described in the discussion, the maximum depth of outer-rise hydration, i.e. serpentinization at deep bending faults, is still a matter of debate.

The link between spreading faults, fault network connectivity, and the lower-plane events is shown in Fig. 4. The upper stereographic projections show a clear distinction in the spatial distribution of faults beneath the Hokkaido and Tohoku regions, with only one or two strike directions, respectively. In the subduction context, faults tend to open parallel to the trench due to the bending of the incoming plate, thereby leading to water percolation and relatively deep serpentinization^10,38,40^. When the bending faults form parallel to the trench, preexisting fabrics or faults may already be present, particularly normal faults associated with oceanic spreading^10,45–47^. When these spreading faults are sub-parallel to the trench, additional bending faults may not be needed due to the easier reactivation and deep propagation of spreading faults^36^. When the spreading faults are not well-oriented, new faults are needed. Abyssal hill fault reactivation is expected to occur beneath the eastern Hokkaido-Oki region^48^ due to the small angle between the Kuril Trench and magnetic isochrones (<10 degrees), which prevents the formation of new faults during the bending of the incoming Pacific Plate. In contrast, that kind of activity may be insufficient beneath the Tohoku-Oki region because of the large angle between the trench axis and magnetic isochrones (54 degrees), so that new faults form in the bending Pacific Plate near the Japan Trench^8,10^. Thus, the higher degree of hydration of the subduction lithosphere beneath the Tohoku region is consistent with the existence of a denser and more connected network (spreading and bending directions) of serpentinized faults.

Since transform faults could be an additional source of fluid within the subducting oceanic plate, further studies should be conducted to investigate their potential link with the b-value variability for the upper-plane beneath the Tohoku region.

### Lower-plane earthquake generation mechanisms at intermediate depths

Based on overall trench geometry^8,10^ and consistency with laboratory experiments^18^, the oceanic mantle is expected to be more hydrated beneath the Tohoku region than beneath the Hokkaido region. For lower-plane events, the higher b-value and lower magnitude beneath the Tohoku region are consistent with the dehydration of a denser and more connected serpentinite network. The DDST^18^ in this network would affect smaller bodies of stressed peridotite, thereby leading to smaller but more numerous earthquakes.

Stress drop studies for intraslab earthquakes beneath the Tohoku^49^ and Hokkaido^50^ regions show that the median stress drops in the oceanic crust and mantle beneath the Tohoku region are smaller than those beneath the Hokkaido region. The median stress drop for lower-plane events is also smaller beneath the Tohoku region than beneath the Hokkaido region. In general, events occur more frequently in highly hydrated rock volumes than in slightly hydrated bodies. Therefore, stress drops associated with events in highly hydrated rocks can be expected to be smaller. The results, and the suggestion of the median stress drops for intermediate-depth intraslab earthquakes are consistent with higher hydration of the subducting oceanic crust beneath the Tohoku region^9^ and with b-values presented in this study (Table 1), i.e. the lower-plane peridotite is more hydrated beneath Tohoku than beneath Hokkaido.

We also considered another hypothesis (thermal shear instabilities)^16,17^ regarding to the occurrence mechanism of oceanic mantle events, which indicated that intermediate-depth oceanic mantle earthquakes occur due to shear heating. In such model, dehydration is not needed in the trigger stage of the occurrence mechanisms, and oceanic mantle events can occur in pure olivine, whereas the DDST model requires dehydration to provide the trigger for strain localization. The thermal shear instabilities hypothesis requires high temperature (more than 600 °C)^17^, but lower-plane events also occur below 600 °C^51^. Based on the thermal structure and seismicity^51^, lower-plane events are located in the temperature range of 400–800 °C. The peak depth of the lower-plane seismicity is located ~30 km from the upper surface of the Pacific Plate beneath the Tohoku and Hokkaido regions^34^. This lower-plane event peak is located in the temperature range of 500 to 600 °C^51^. On the other hand, unlike thermal shear instabilities, the occurrence mechanism proposed by the DDST model is compatible with temperatures of 550 to 700 °C. Hence, the DDST model better explains the seismicity peak of lower-plane events beneath northeastern Japan. Nevertheless, the thermal shear instabilities hypothesis remains another possible occurrence mechanism.

### Interplane events, upper-plane events and fluid overpressure

The b-values for interplane events beneath the Tohoku and Hokkaido regions are low and almost the same, which indicates that seismic events are much more localized, suggesting that interplane earthquakes do not have the same triggering mechanism, i.e. the DDST model would only apply for the lower-plane earthquakes. Alternatively, dehydration embrittlement, both in the upper plane and in between planes, is likely to be fluid-induced^27,51–55^. Increasing fluid pressure in preexisting faults would locally reduce their shear strength and trigger instability^24,53,56^, unlike lower-plane events where new faults would dynamically propagate in fresh peridotite at the tip of dehydrating antigorite clusters^18^.

The normal distance range of interplane events is 10 to 23 km from the subduction interface beneath both the Hokkaido and Tohoku regions^34^. These earthquakes are observed to be shallower than the maximum depth of the outer-rise fault activity^37,57,58^, i.e. <~40 km (compressional outer-rise events and intraplate events)^59,60^, at temperatures <600 °C^61^, i.e. near the antigorite stability limit. A very precise study using sP delay times beneath the Tohoku outer-rise region^57^ shows that the outer-rise extensional events are located shallower than 20 km from the surface of the Pacific Plate, while outer-rise compressional events are located at depths from 32 to 43 km^57^. This indicates that the depth range of interplane events from the subduction interface is included in the depth range of the outer-rise extensional events. Hence, interplane events could occur in peridotite that underwent outer-rise extensional faulting. Indeed, extensional faulting would lead to dense serpentinization at faults and/or dense fault networks. As a consequence, the interplane events should have triggering mechanisms that are similar to the events occurring in the oceanic crust, i.e. fluid-related^34^. On the other hand, there is not enough antigorite in the lower-plane of the double seismic zone to induce fluid overpressure upon dehydration, especially when P > 1.5 GPa because overpressure is impossible due to negative volume change^18,53^. Thus, earthquakes are more readily explained by the DDST in the lower plane.

As upper-plane b-values show strong variations between three subareas in the Tohoku region, further studies are required to interpret the results for upper-plane events in detail. Furthermore, since various hydration processes exist in the oceanic crust within the subducting oceanic plate and would lead to such a variety of b-values, further seismological and experimental studies are required to better understand the variations in earthquake distributions beneath the Tohoku region.

### Effect of the ***M***9 event

We compared the b-values for intraslab events beneath the Tohoku region before and after the *M*9 event and confirmed the decrease in b-values for upper-plane events after the *M*9 earthquake as well as a slight decrease for interplane and lower-plane events. This general decrease in b-values indicates a more localized seismicity after the *M*9 event regardless of type, which implies that the stress states at upper- and lower-plane positions are somehow connected. Nevertheless, the drop in b-values is not that large, which means that the event populations at depths of 60 to 200 km beneath the inland Tohoku region were largely unchanged by the *M*9 event. Critical stress intensity triggering an intermediate-depth earthquake would be localized around the hypocenter region.

The intraslab seismic activity at intermediate depths beneath the Tohoku region before and after the *M*9 event indicates that the ratio of upper-plane to lower-plane events increased significantly just after the *M*9 event^62^. Taking these results into consideration, we argue that the larger drop in b-value for upper-plane events in this study could be related to easier stress transfer in the upper plane than in the lower plane.

### Conclusions and seismological validation of the DDST model

Calculating b-values for intermediate-depth seismicity within the Pacific slab beneath northeastern Japan reveals their spatial variation within the slab beneath the Tohoku and Hokkaido regions.

For the lower-plane events, the b-value beneath Tohoku (0.96) is larger than that beneath the Hokkaido region (0.86). According to the comparison with experimental b-values, a possible interpretation of this difference is that the higher b-value beneath the Tohoku region than beneath the Hokkaido region corresponds to a larger population of deep serpentinized faults. This greater outer-rise hydration of the oceanic mantle near the Japan Trench could be related to a denser and more connected fault network that is caused by the high angle between the spreading faults and the bending faults (parallel to the strike of the Japan trench). In contrast, the bending faults near the Kuril Trench are sub-parallel to the spreading faults, which limits the fault density and connectivity and promotes larger and more localized earthquakes.

A denser fault network leads to a denser serpentinization, which reduces the size of fresh peridotite bodies and thus the average size of potential dehydration-driven earthquakes. Following the DDST model, lower-plane earthquakes could be triggered by a dehydration-driven stress transfer at the tip of dehydrating serpentine clusters. These earthquakes would dynamically propagate in fresh peridotite between serpentinized faults, which would be consistent with several experimental studies^23,25,63,64^.

For interplane events, consistently smaller b-values both beneath the Tohoku (0.71) and Hokkaido (0.74) regions indicate more localized seismic activity. Although both interplane and lower-plane events occur within the oceanic mantle, their occurrence mechanisms are different. Both upper-plane and interplane events possibly occur by “dehydration embrittlement”, meaning they are fluid-related. More specifically, increasing fluid pressure in preexisting faults would locally reduce their shear strength and trigger instability. Because not all large faults reach the interplane zone, fresh rock volumes are larger at upper-plane locations, which means that relatively larger events can occur in the interplane zone than in the upper plane.

This study could be a step towards an improved understanding of intermediate-depth earthquake physical mechanisms, and towards creating more precise strong motion prediction in the future, which could then help reduce intraslab earthquake hazards.

## Methods

### Estimation of b-values

The b-value is the slope of a Gutenberg-Richter distribution. For a given number of earthquakes, a large b-value means that $\bar{M}$ tends to be smaller, whereas a small b-value means that relatively larger events occur.

For the b-value calculation, we adopted^65^:

$$b=\frac{{\mathrm{log}}_{10}e}{\bar{M}-{M}_{c}}\,,$$

where log_10_*e* = 0.43429, *M*_c_ is the complete magnitude (i.e. the lower limit of the detected magnitude) and $\bar{M}$ is the average magnitude for events for which *M* > *M*_*c*_. As previously reported, *M*_*c*_ = *M*_JMA_ = 1.2 for intraslab earthquakes beneath the Tohoku and Hokkaido regions^66^. In Figs. 2a and b, *M*_c_ = 1.2 seems to be appropriate, but we calculated b-values using *M*_*c*_ = 1.2, 1.3, 1.4, 1.5, 1.6, 1.7, 1.8, 1.9, 2.0 and 2.1, as shown in Fig. S2. Finally, we adopted *M*_*c*_ = *M*_JMA_ = 1.9 in this analysis because all the b-values for the upper-plane, interplane and lower-plane events are stable for *M*_c_ >1.6 beneath the Hokkaido region and *M*_*c*_ > 1.8 beneath the Tohoku region. We also calculated *R*, the goodness-of-fit^67^. The *R* values for all kinds of intraslab events beneath the Tohoku and eastern Hokkaido regions are over 90% with *M*_*c*_ = 1.7, 1.8 and 1.9 beneath the Tohoku region and *M*_c_ = 1.6, 1.7, 1.8 and 1.9 beneath the Hokkaido region. As *M*_c_ values are reliable if *R* > 90%^68^, the *M*_c_ value in this study is reasonable. Furthermore, this value is close to a previous estimation *M*_c_ ≈ 2.0 for intraslab events beneath the Tohoku-Oki region^69^. The 95% confidence level for b-value estimations is given as $b/\surd N$ (*N*: number of events)^70^.

### Relocation procedure

We relocated intraslab events using the double difference relocation method^30^, assuming the routine velocity structure of Tohoku University^11^ and hypocenter parameters from the dense nationwide seismic network^71,72^. Arrival data from the Japan Meteorological Agency (JMA) catalog were used in the relocation process. Relocation estimation mean errors are about 1 km based on results obtained by using the singular value decomposition (SVD) method^30^. Because of computer capacity limitations, the study area was divided into five sub-areas (Table S1) and hypocenters in each subarea were relocated (Fig. S1).

In all, 86,655 events were relocated using the double difference relocation method in and above the subducted Pacific slab. Since sub-region (b)’ shown in Fig. S1 overlapped with sub-regions (a) and (c), we examined relocated events in sub-region (b). We defined sub-region (e) in the east coast of central Tohoku region, because the 2003 Miyagi intraslab event (M_jma_ 7.1) and its aftershocks occurred frequently. In total, 6,856,295 arrival time differences were obtained from catalog data for P-waves, and 5,341,984 arrival time differences were obtained from the data for S-waves. Event pairs were selected that had hypocentral separations of less than 20 km and more than eight arrival time differences with respect to their neighbors. The final results of the inversion were obtained after 18 iterations. The average root mean square residuals of the double differences were reduced after the relocations. Detailed relocation results are summarized in Table S1.

### Calculation of experimental b-values

In total, 79 AEs were recorded in dehydrating antigorite-olivine aggregates^18^. Here, we use the data from samples deformed at 3.5 GPa (≈120 km), which is the pressure at the middle of the lower-plane events. In those pressure-temperature conditions (Fig. 3c), laboratory earthquakes were triggered with different volume fractions of antigorite (Fig. 3a), i.e. the high-temperature serpentine variety.

The magnitude of an acoustic emission was calculated as: *M*_AE_ = log_10_*E*, with *E* taken as the average energy (V^2^) of the amplified acoustic signals. This quantity is relative, like the magnitude defined for earthquakes, and permits one event to be compared with another. The frequency of relative moment magnitudes (*M*_AE_) follows a Gutenberg-Richter (GR) distribution (log_10_*N* = *a *− *bM*_AE_) (Fig. 3e) with different b-values depending on the antigorite fraction (Tables S3, S4, and S5, Figs. 3e and 3f). For each experiment, several calculations were performed with various M_AE_ thresholds (magnitude of completeness). Absolute values of both *M*_AE_ and *b* are not comparable with natural data. For instance, these laboratory earthquakes, with 0 < *M*_AE_ < 3, have moment magnitudes *M*_w_ ranging between −7 and −6. Nevertheless, their evolution must be comparable. Here, while *M*_AE_ decreases with increasing antigorite fraction (Fig. 3e), *b* increases, showing a near-linear relationship (Fig. 3f). The colored areas in Fig. 3e highlight the difference between the two thresholds, i.e. for *M*_AE_ > 0 and for *M*_AE_ > 0.5.

## Electronic supplementary material

Supplementary file
