# Supplementary material for: Physical mechanisms of oceanic mantle earthquakes: Comparison of natural and experimental events
Source: Sci Rep. 2018 Nov 19;8:17049. doi: 10.1038/s41598-018-35290-x (PMC6242829; doi:10.1038/s41598-018-35290-x)
Supplement: Supplementary file 1 — Supplementary file [file 41598_2018_35290_MOESM1_ESM.docx]

# Supplementary information

# Physical mechanisms of oceanic mantle earthquakes: Comparison of natural and experimental events

Saeko Kita^1, 2^, Thomas P. Ferrand^3,4^

1: Graduate School of Science, Hiroshima University, Kagamiyama1-3-1, Higashi-Hiroshima, 739-8526, Japan;

2: Building Research Institute, Tatehara 1, Tsukuba, 305-0802, Japan

3: Laboratoire de Géologie – UMR 8538, CNRS, Ecole Normale Supérieure, PSL University, 75005 Paris, France;

4: Earthquake Research Institute, University of Tokyo, 1-1-1 Yayoi, Bunkyo-ku, Tokyo 113-0032, Japan.

Corresponding author: Saeko Kita – *saeko@hiroshima-u.ac.jp*

**Tables:**

|  | | (a) | (b) | (c ) | (d) | (e ) |
| --- | --- | --- | --- | --- | --- | --- |
| Nb. of events for relocation | | 27,990 | 14,976 | 13,338 | 16,482 | 22,907 |
| Nb. of stations | | 111 | 132 | 141 | 236 | 53 |
| Nb. of DDｓ(from ct) | P waves | 1,738,402 | 924,117 | 1,012,155 | 1,168,244 | 2,013,377 |
|  | S waves | 1,257,344 | 477,824 | 768,588 | 919,222 | 1,919,006 |
| DDs RMS (s): | before | 0.213 | 0.231 | 0.1439 | 0.165 | 0.113 |
|  | after | 0.0594 | 0.0729 | 0.0615 | 0.0617 | 0.0538 |

**Table S1:** Datasets used in the relocation process. The five sub-regions **[OK?]** are shown in **Fig. S1**. For each of them the number of events and stations, as well as the number of the double-differences (DDs) for P and S waves, are indicated. The relocation process leads to a reduction of the root mean square (RMS) of the DDs between two events. Residuals correspond to the difference between calculated and observed arrival times.

|  | Tohoku (before M9) | | | | | | | | |
| --- | --- | --- | --- | --- | --- | --- | --- | --- | --- |
|  | North (more than 40 °N) | | | Central (38.5-40°N) | | | South (less than 38.5°) | | |
| Location | b-value | | nb EQs | b-value | | nb EQs | b-value | | nb EQs |
| Entire slab | 0.84 | ± 0.028 | 866 | 0.79 | ±0.013 | 3975 | 0.97 | ±0.024 | 1581 |
| Upper plane | 0.78 | ± 0.040 | 385 | 0.84 | ±0.021 | 1645 | 1.03 | ±0.043 | 578 |
| Interplane | 0.76 | ± 0.054 | 199 | 0.74 | ±0.017 | 1864 | 0.80 | ±0.045 | 316 |
| Lower plane | 0.99 | ± 0.057 | 302 | 0.92 | ±0.042 | 467 | 0.99 | ±0.038 | 687 |

**Table S2:** Calculated b-values and associated errors for small eastern Hokkaido and Tohoku sub-regions, using M_c=1.9. (a) Results for three Tohoku sub-regions.

|  | Tohoku (before M9) | | | | | |
| --- | --- | --- | --- | --- | --- | --- |
|  | South (less than 39 °N) | | | North (more than 39°N) | | |
| Location | b-value | | nb EQs | b-value | | nb EQs |
| Entire slab | 0.82 | ±0.012 | 4934 | 0.85 | ±0.022 | 1508 |
| Upper plane | 0.87 | ±0.019 | 2037 | 0.84 | ±0.035 | 571 |
| Interplane | 0.74 | ±0.016 | 2056 | 0.79 | ±0.044 | 323 |
| Lower plane | 1.01 | ±0.035 | 842 | 0.92 | ±0.037 | 614 |

**Table S2** (continued): (b) Results for two Tohoku sub-regions.

|  | E-Hokkaido | | | | | |
| --- | --- | --- | --- | --- | --- | --- |
|  | West (less than 144 °E) | | | East (more than 144°E) | | |
| Location | b-value | | nb EQs | b-value | | nb EQs |
| Entire slab | 0.84 | ±0.021 | 1651 | 0.89 | ±0.021 | 1779 |
| Upper plane | 1.01 | ±0.056 | 330 | 0.97 | ±0.045 | 468 |
| Interplane | 0.69 | ±0.039 | 309 | 0.72 | ±0.044 | 271 |
| Lower plane | 0.85 | ±0.027 | 1012 | 0.90 | ±0.028 | 1040 |

**Table S2** (continued): (c) Results for two eastern Hokkaido sub-regions.

|  | E-Hokkaido | | | | | |
| --- | --- | --- | --- | --- | --- | --- |
|  | West (less than 143.5 °E) | | | East (more than 143.5°E) | | |
| Location | b-value | | nb EQs | b-value | | nb EQs |
| Entire slab | 0.84 | ±0.027 | 929 | 0.87 | ±0.017 | 2501 |
| Upper plane | 0.99 | ±0.076 | 169 | 0.99 | ±0.039 | 629 |
| Interplane | 0.69 | ±0.052 | 176 | 0.71 | ±0.035 | 404 |
| Lower plane | 0.85 | ±0.035 | 584 | 0.89 | ±0.023 | 1468 |

**Table S2** (continued): (d) Results for two eastern Hokkaido sub-regions.

| Antigorite fraction  (vol. %) | | 0 | 5 | 20 | 50 |
| --- | --- | --- | --- | --- | --- |
| b-value | M_AE_ > 0 | 0.33 | 0.37 | 0.53 | 0.48 |
|  | M_AE_ > 0.25 | 0.33 | 0.44 | 0.48 | 0.58 |
|  | M_AE_ > 0.5 | 0.32 | 0.42 | 0.44 | 0.59 |
|  | M_AE_ > 0.75 | 0.30 | 0.45 | 0.37 | 0.62 |

**Table S3:** Experimental b-values using different M_AE_ thresholds, part of which is shown in Fig. 3e. See explanations in the dedicated paragraph of the Methods Section.

| Antigorite fraction  (vol. %) | | 0 | 5 | 20 | 50 |
| --- | --- | --- | --- | --- | --- |
| Total nb. events | M_AE_ > 0 | 9 | 12 | 16 | 14 |
|  | M_AE_ > 0.25 | 9 | 10 | 10 | 14 |
|  | M_AE_ > 0.5 | 8 | 10 | 7 | 13 |
|  | M_AE_ > 0.75 | 6 | 10 | 4 | 11 |

**Table S4:** Number of events within serpentinized peridotite samples using different M_AE_ thresholds.

| Antigorite fraction  (vol. %) | | 0 | 5 | 20 | 50 |
| --- | --- | --- | --- | --- | --- |
| R^2^ | M_AE_ > 0 | 0.898 | 0.934 | 0.929 | 0.897 |
|  | M_AE_ > 0.25 | 0.870 | 0.946 | 0.913 | 0.920 |
|  | M_AE_ > 0.5 | 0.835 | 0.951 | 0.897 | 0.915 |
|  | M_AE_ > 0.75 | 0.755 | 0.946 | 0.858 | 0.876 |

**Table S5**: Values of R^2^ (determination coefficient) for serpentinized peridotite samples using different M_AE_ thresholds.

## Figure:

**Figure S1:** Sub-regions used in the relocations. Black lines show sub-regions (a), (b), (c), (d) and (e), which were adopted for calculations in this study. Dashed line shows sub-region (b’), in which the calculation is performed for events in sub-region (b), i.e. events in sub-region (b’) are compiled only once into the relocated dataset, as part of (a) and (c).


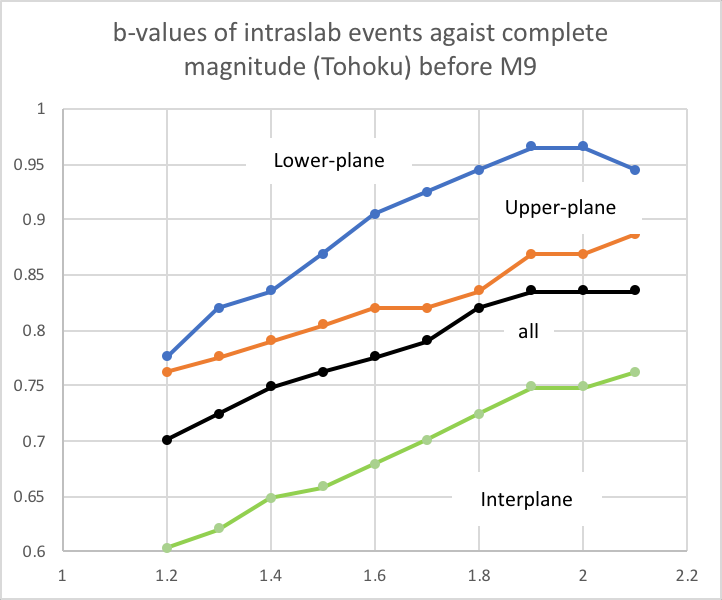

**Figure S2:** (a) (b) b-values of intraslab events against complete magnitude beneath (a) Tohoku and (b) eastern Hokkaido.
